# Tree‐drawing test characteristics as potential predictors of adolescent depressive disorders

**DOI:** 10.1002/ibra.70030

**Published:** 2026-09-22

**Authors:** Yibin Lu, Xutong Chen, Qiqige, Rong Yi, Jike Bai

**Affiliations:** ^1^ Department of Child and Adolescent Psychology Dongguan Mental Health Center of Southern Medical University (Dongguan Seventh People's Hospital) Dongguan China; ^2^ Department of Clinical Psychology Dongguan Qingxi Hospital Dongguan China; ^3^ The Third People's Hospital of Longgang District Clinical Institute of Shantou University Medical College Shenzhen China; ^4^ School of Interpreting and Translation Studies Guangdong University of Foreign Studies Guangzhou China

**Keywords:** adolescent depressive disorders, projective tests, screening, Tree‐Drawing Test

## Abstract

This study examined the relationship between adolescent depressive disorders and drawing characteristics identified in the Tree‐Drawing Test (TDT) and developed a predictive model based on TDT features. Participants included 48 adolescents diagnosed with depressive disorders and 32 healthy controls. All participants completed the TDT and the Self‐Rating Depression Scale (SDS). Group differences in drawing characteristics were analyzed using chi‐square tests. Binary logistic regression analysis was conducted with depressive disorder status (presence vs. absence) as the dependent variable and selected drawing characteristics as independent variables to identify significant predictors. Significant differences between the depression and control groups were found across 10 drawing characteristics. Logistic regression analyses indicated that tree shading or darkened areas (odds ratio [OR] = 4.155, *p* = 0.045), overly detailed depiction of the tree (OR = 7.094, *p* = 0.007), and disorganized crown lines (OR = 7.828, *p* = 0.003) were potential independent predictors of adolescent depressive disorders. A logistic model incorporating these three characteristics achieved an overall classification accuracy of 83.8% and an area under the receiver operating characteristic curve (AUC) of 0.900. These findings provide preliminary evidence that the TDT may serve as a useful supplementary tool for the assessment of depressive disorder in adolescents. Further validation in larger and independent samples is required.

## Graphical abstract

Among 12 Tree‐Drawing Test (TDT) characteristics, 10 characteristics differed significantly between depressive and control groups. Tree shading, excessive detail, and disorganized crown lines independently predicted depressive disorders. A three‐feature logistic model achieved 83.8% classification accuracy and an AUC of 0.900, supporting TDT as a promising supplementary assessment tool for adolescent depressive disorders.

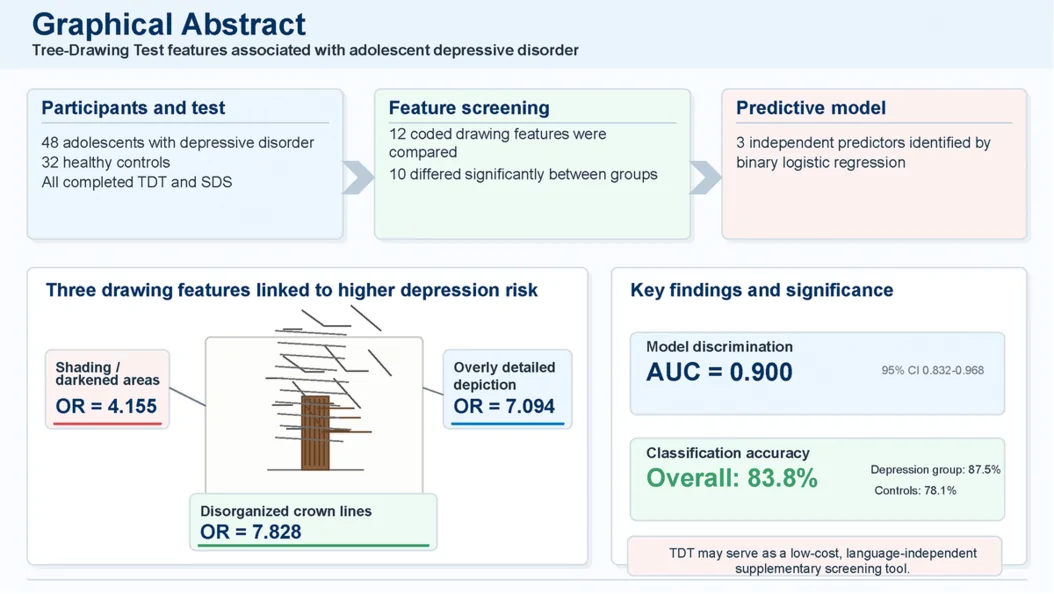

## 1 INTRODUCTION

Depressive disorder is one of the most common mental disorders among adolescents. Its core symptoms include persistent low mood, loss of interest or pleasure, decreased energy, psychomotor retardation, and sleep disturbances; in severe cases, it may lead to self‐harm and suicidal behaviors.^1^ In recent years, the prevalence of adolescent depressive disorder has shown a steady upward trend.^2^ According to reports from the World Health Organization, depression and anxiety have become leading causes of disability among individuals aged 10–19 years worldwide, while suicide ranks among the leading causes of death in the 15–29‐year age group.^3^ Epidemiological studies likewise indicate that the detection rate of depressive disorder among adolescents is substantial. Survey data suggest that the lifetime prevalence of depressive disorder in China is approximately 3.4%, with a continuing upward trend in prevalence rates.^4^ Adolescent depressive disorder exerts profound negative effects on academic performance, interpersonal relationships, and social functioning, and it also imposes a considerable burden on public health systems.^5^ Therefore, the early identification and intervention of depressive disorder in adolescents are of significant clinical and social importance.

Despite the availability of effective treatments for depression, up to 80% of adolescents with depressive disorder do not receive adequate care.^6^ In primary care settings, among adolescents newly identified with depressive symptoms, 36% receive no treatment within 3 months, and 68% do not undergo follow‐up symptom assessment (e.g., using the Patient Health Questionnaire [PHQ]). More concerningly, even among adolescents who are prescribed antidepressant medication, 40% have no documented follow‐up visit within 3 months after prescription initiation.^6^ Given the continued rise in adolescent suicide rates, these deficiencies in the quality of care have raised serious concerns regarding the management of adolescent depression_._ To address these gaps, quality improvement initiatives have demonstrated that the implementation of universal depression screening in primary care settings (e.g., using the PHQ‐A) is feasible and can significantly increase rates of depression diagnosis (e.g., from 0% to 12.1%) as well as referrals to mental health services (8%).^7^ Once high‐risk adolescents are identified, interventions such as Mind–Body Skills Groups have been shown to be acceptable and effective, leading to significant improvements in depressive symptoms, mindfulness, and self‐efficacy among adolescents.^8^

In the diagnosis and screening of depressive disorder, self‐report measures (e.g., the PHQ‐9 and the Self‐Rating Depression Scale [SDS]) are widely used. Although these instruments are brief and easy to administer, their accuracy may be compromised by individuals' subjective responses, cultural background, and social desirability bias, potentially resulting in insufficient sensitivity and specificity.^9^, ^10^ Therefore, the exploration of assessment methods that do not rely on language and that more directly reflect underlying psychological states is of considerable importance for improving the objectivity and effectiveness of depression screening. The Tree‐Drawing Test (Koch's Tree‐Drawing Test, TDT) is a classic projective drawing technique originally proposed by Koch. Owing to its simplicity, minimal language dependence, and strong cross‐cultural applicability, the TDT has been widely used in psychological assessment among children and adolescents.^11^ Previous research has demonstrated that projective drawing tests can reveal individuals' deeper psychologicalexperiences and personality characteristics^12^ and have shown utility in the screening of mental disorders and the assessment of trauma‐related responses.^13^ Moreover, the TDT that can be analyzed not only qualitatively but also through objective quantitative indices to assist clinical diagnosis.^14^ Gu et al.^15^ quantified geometric features of tree drawings in adults with depression, whereas evidence based on clinically observable drawing characteristics in adolescents remains limited. Therefore, the present study examined clinically observable TDT characteristics in adolescents with depressive disorder and explored their associations with diagnostic status.

## 2 METHODS

### 2.1 Study design

This study employed a cross‐sectional observational design to examine differences in TDT characteristics between adolescents with depressive disorder and healthy controls, as well as to explore the psychological significance of these drawing features.

### 2.2 Participants and inclusion criteria

All participants were recruited from Dongguan City, Guangdong Province, China. The diagnosis was independently confirmed by two or more attending psychiatrists according to the DSM‐5 diagnostic criteria. This study was approved by the Ethics Committee of Dongguan Seventh People's Hospital (Approval No. 2022‐003). Written informed consent was obtained from all participants and their legal guardians prior to participation.

#### 2.2.1 Depression group

The depression group was recruited using convenience sampling from a mental health center in Dongguan City. Participants were adolescents who met the diagnostic criteria for depressive disorder according to the Diagnostic and Statistical Manual of Mental Disorders (5th ed.; DSM‐5). Inclusion criteria were as follows: (a) age between 10 and 18 years; (b) meeting DSM‐5 diagnostic criteria for depressive disorder; (c) voluntary participation in the study; and (d) clear consciousness and a basic level of education. Exclusion criteria included: (a) age over 18 years; (b) comorbid psychiatric disorders; (c) severe depressive disorders accompanied by suicidal behavior or stupor; and (d) severe physical illness or organic brain disease. All assessments were conducted individually in a quiet psychotherapy room.

#### 2.2.2 Control group

The control group was recruited through volunteer enrollment from a primary and secondary school in Dongguan City. Inclusion criteria were: (a) not meeting DSM‐5 diagnostic criteria for depressive disorder; (b) voluntary participation; and (c) clear consciousness and a basic level of education. Exclusion criteria included: (a) age over 18 years; (b) history of psychiatric disorders; (c) severe physical illness or organic brain disease; and (d) an SDS standard score of 53 or higher. Participants in the control group completed the TDT collectively in a school counseling office.

### 2.3 Measures

#### 2.3.1 TDT

TDT, originally developed by Koch, was used to assess participants' psychological characteristics and life experiences. During the assessment, participants were given the following standardized instruction: “Please draw a fruit‐bearing tree. This is not a test of drawing ability. You do not need to focus on esthetic quality or realism. Simply draw the tree you most want to draw. Before you begin, please close your eyes and meditate for 30 s, and then start drawing.” The time limit for drawing was 8 min.

Based on Koch's TDT framework and previous clinical research, two trained clinicians independently evaluated the drawings and identified 12 drawing characteristics, each of which was operationally defined. All drawing characteristics were coded as binary variables, with the presence of a feature coded as 1 and its absence coded as 0. Detailed operational definitions of these characteristics are presented in Table 1. To ensure measurement reliability, all drawing characteristics were independently rated by two evaluators who were blinded to participants' group assignments.

**Table 1:** Drawing characteristics and variable coding.

Factor	Variable	Coding description
Presence of depressive disorder	Y	0 = No, 1 = Yes
Tree shading or darkened areas	X1	0 = Absent, 1 = Present
Trembling or shaky lines	X2	0 = Absent, 1 = Present
Small tree size	X3	0 = Tree height > 1/3 of paper height; 1 = Tree height < 1/3 of paper height
Tree positioned in lower part of page	X4	0 = Tree center in upper half of page; 1 = Tree center in lower half
Overly detailed depiction of the tree	X5	0 = Absent, 1 = Present
Disorganized lines within the crown	X6	0 = Absent, 1 = Present
Tree positioned in upper part of page	X7	0 = Absent, 1 = Present
Whole tree leaning to the left	X8	0 = Absent, 1 = Present
Scars or stains on the trunk	X9	0 = Absent, 1 = Present
Irregular branches on the left side of the crown	X10	0 = Absent, 1 = Present
Sharp, knife‐like branches	X11	0 = Absent, 1 = Present
Presence of fruit	X12	0 = Absent, 1 = Present

*Note*: All drawing characteristics were coded as binary variables for statistical analysis.

#### 2.3.2 Depression measure

Depressive symptoms were assessed using the Self‐Rating Depression Scale (SDS) developed by Zung.^16^ The SDS consists of 20 items rated on a 4‐point Likert scale, an evaluation completed within 10 min, yielding a total score ranging from 20 to 80, with higher scores indicating greater severity of depressive symptoms. According to the Chinese scoring criteria described in the *Manual of Mental Health Rating Scales*, a standard score of 53 or higher was used as the cutoff for an elevated possibility of depressive disorders. In the present study, the SDS was used to assess the severity of depressive symptoms, whereas the diagnosis of depressive disorder was based on the DSM‐5 criteria.

### 2.4 Data collection and statistical analysis

All data were independently entered by two researchers using Microsoft Excel and subsequently cross‐checked for accuracy. Statistical analyses were conducted using SPSS version 26.0. Descriptive statistics were used to summarize demographic and clinical characteristics. Group differences were examined using chi‐square tests and independent‐samples *t* tests. Binary logistic regression analysis was performed to explore the associations between drawing characteristics and depressive disorder. Model goodness of fit was evaluated using the Hosmer–Lemeshow test. The *p* values less than 0.05 were considered statistically significant.

## 3 RESULTS

### 3.1 Participant characteristics

A total of 80 participants were recruited, including 48 participants in the depression group and 32 in the control group. The mean age of participants in the depression group was 14.13 years (SD = 2.11), which comprised 19 males and 29 females, and 14.81 years (SD = 1.71) in the control group, which comprised 13 males and 19 females. No significant differences were found between groups in age or gender (Table 2).

**Table 2:** Comparisons of age and gender between the depression group and the control group.

Variable	Depression group (*n* = 48)	Control group (*n* = 32)	*t*/*χ* ^2^	*p*
Age (years), *M* ± SD	14.13 ± 2.11	14.81 ± 1.71	2.359	0.129
Male, *n* (%)	19 (39.58%)	13 (40.63%)	0.009	0.926
Female, *n* (%)	29 (60.42%)	19 (59.37%)		

*Note*: M ± SD = mean ± standard deviation. Age differences were examined using independent‐samples t tests, and gender differences were examined using chi‐square tests. No significant differences were observed between the two groups (*p* > 0.05).

### 3.2 Interrater reliability

A total of 80 drawings were collected. To ensure the reliability of the evaluation of drawing characteristics, two independent trained researchers conducted blinded ratings of the 12 predefined TDT features in adolescents with depressive disorder. Statistical analyses indicated that the kappa coefficients for all drawing characteristics exceeded 0.861, demonstrating a high level of interrater agreement and good reliability of the ratings.

### 3.3 Distribution of drawing characteristics and group difference

Among the 12 drawing features, 10 features showed significant differences between the depressed and control groups (Table 3). Compared with the control group, the drawing from depressed group more frequently exhibited the following features: trees with shading or dark areas (64.58% vs. 18.75%, *χ* ^2^ = 16.23, *p* < 0.001), shaky tree lines (77.08% vs. 12.50%, *χ* ^2^ = 32.05, *p* < 0.001), small trees (77.08% vs. 9.38%, *χ* ^2^ = 35.21, *p* < 0.001), trees located at the bottom of the paper (52.08% vs. 6.25%, *χ* ^2^ = 18.04, *p* < 0.001), trees drawn with detailed strokes (64.58% vs. 12.50%, *χ* ^2^ = 21.16, *p* < 0.001), disordered lines in the tree crown (79.17% vs. 18.75%, *χ* ^2^ = 28.32, *p* < 0.001), tree trunks with scars or spots (79.17% vs. 15.63%, *χ* ^2^ = 33.41, *p* < 0.001), irregular branches on the left side of the crown (31.25% vs. 0.0%, *χ* ^2^ = 12.31, *p* < 0.001), sharp, knife‐like branches (35.42% vs. 6.25%, *χ* ^2^ = 9.02, *p* = 0.003), and trees with fruit (8.33% vs. 28.13%, *χ* ^2^ = 5.53, *p* = 0.019).

**Table 3:** Comparison of tree drawing features between the depressed and control groups.

Feature	Depressed group (*n* = 48)	Control group (*n* = 32)	*χ* ^2^	*p*
Trees with shading or dark areas	31 (64.58%)	6 (18.75%)	16.23	<0.001
Shaky tree lines	37 (77.08%)	4 (12.50%)	32.05	<0.001
Small trees	37 (77.08%)	3 (9.38%)	35.21	<0.001
Trees at the bottom of the paper	25 (52.08%)	2 (6.25%)	18.04	<0.001
Trees drawn with detailed strokes	31 (64.58%)	4 (12.50%)	21.16	<0.001
Disordered lines in tree crowns	38 (79.17%)	6 (18.75%)	28.32	<0.001
Tree trunks with scars or spots	38 (79.17%)	5 (15.63%)	33.41	<0.001
Irregular branches on left side of crown	15 (31.25%)	0 (0.00%)	12.31	<0.001
Sharp, knife‐like branches	17 (35.42%)	2 (6.25%)	9.02	0.003
Trees located at the top of the paper	7 (14.58%)	0 (0.00%)	3.45	0.063
Whole tree shifted to the left	8 (16.67%)	3 (9.38%)	0.36	0.551
Trees with fruit	4 (8.33%)	9 (28.13%)	5.53	0.019

*Note*: *χ* ^2^ = chi‐square statistic; *p* = significance level.

In contrast, “trees located at the top of the paper” (14.58% vs. 0.0%, *χ* ^2^ = 3.45, *p* = 0.063) and “whole tree shifted to the left” (16.67% vs. 9.38%, *χ* ^2^ = 0.36, *p* = 0.551) did not differ significantly between the two groups.

### 3.4 Logistic regression analysis: The association between drawing features and depressive disorders

Drawing features that were statistically significant in the preliminary difference tests were included in a binary logistic regression model, with depressive disorder (presence/absence) as the dependent variable. Due to sample size limitations, only the variables with the best model fit were included in the regression equation.

#### 3.4.1 Model fit and explained variance

The overall chi‐square test of the model indicated that the regression model was significantly better than the null model (*χ* ^2^ = 47.044, *p* < 0.001), suggesting that the model was statistically significant. The ‐2 log‐likelihood value was 60.638, Cox & Snell *R* ^2^ = 0.445, and Nagelkerke *R* ^2^ = 0.601, indicating a high model fit. The Hosmer–Lemeshow goodness‐of‐fit test yielded *p* = 0.611 (*p* > 0.05), further supporting the adequacy of the model fit (Table 4).

**Table 4:** Fit indices of the logistic regression model.

Statistic	Value
Chi‐Square	47.044
*p*	0.001
−2 Log‐Likelihood	60.638
Cox & Snell *R* ^2^	0.445
Nagelkerke *R* ^2^	0.601
Hosmer–Lemeshow Test *χ* ^2^	5.404
df	7
*p*	0.611

*Note*: The Hosmer–Lemeshow test indicates good model fit when *p* > 0.05.

#### 3.4.2 Key predictors

Logistic regression analysis indicated that three variables were significantly associated with depressive disorders: trees with shading or darkened areas (OR = 4.155, 95% CI [1.031–16.745], *p* = 0.045), trees depicted in detailed manner (OR = 7.094, 95% CI [1.693–29.733], *p* = 0.007), and disordered lines within the tree canopy (OR = 7.828, 95% CI [2.038–30.070], *p* = 0.003) (Table 5). Among these, disordered lines within the tree canopy had the largest effect size, followed by trees depicted in a detailed manner, while shading or darkened areas had the smallest effect. Other variables were not statistically significant.

**Table 5:** Logistic regression analysis of drawing features and adolescent depressive disorders.

Variable	B	SE	Wald	*p*	OR	95% CI
Trees with shading or darkened areas (X1)	1.424	0.711	4.012	0.045	4.155	1.031–16.745
Trees depicted in detailed manner (X5)	1.959	0.731	7.182	0.007	7.094	1.693–29.733
Disordered lines within tree canopy (X6)	2.058	0.687	8.981	0.003	7.828	2.038–30.070

*Note*: *p* values are two‐tailed.

Abbreviations: B = regression coefficient, CI = confidence interval, OR = odds ratio, SE = standard error, Wald = Wald statistic.

#### 3.4.3 Predictive model and accuracy

The model was constructed based on the regression coefficients as follows:

$$\text{Logit}(P)=-1.714+1.424X_{1}+1.959X_{5}+2.058X_{6}.$$

Using 0.5 as the cutoff point for prediction, the model correctly predicted 87.5% (42/48) of cases in the depressive group and 78.1% (25/32) in the normal group, with an overall accuracy of 83.8%, indicating satisfactory predictive performance.

The receiver operating characteristic (ROC) analysis showed that the area under the curve (AUC) of the model was 0.900 (95% CI [0.832–0.968], *p* < 0.001), indicating strong discriminatory ability (Figure 1; Table 6). Combined analysis of sensitivity and specificity indicated that the optimal cutoff point was 0.512, corresponding to the maximum Youden index of 0.656.

**Figure 1:**
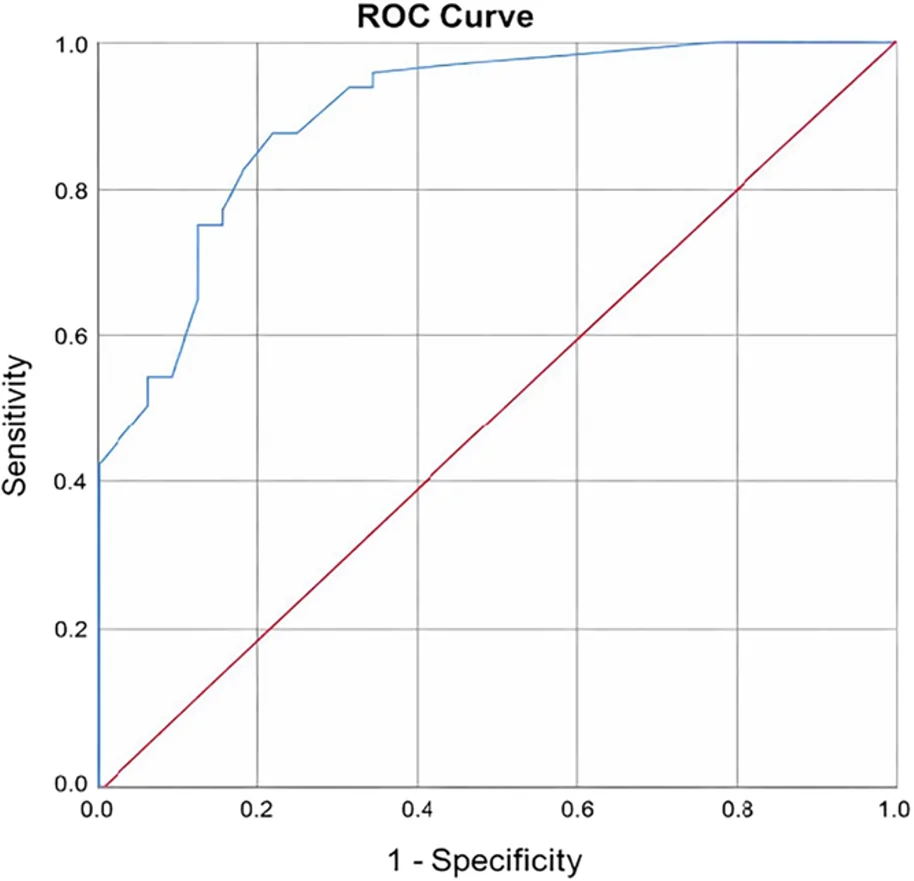
ROC curve of the logistic model predicting depressive disorders based on drawing features. ROC, receiver operating characteristic.

**Table 6:** Results of ROC curve analysis.

Measure	AUC	Standard error	Asymptotic significance	95% CI lower	95% CI upper
ROC curve	0.900	0.035	0.001	0.832	0.968

*Note*: Standard error is based on nonparametric assumptions. The null hypothesis assumes the true area = 0.5.

Abbreviations: AUC, area under the curve; CI, confidence interval; ROC, receiver operating characteristic.

## 4 DISCUSSION

Unlike Gu et al.,^15^ who mainly examined continuous geometric indices in an adult sample, the present study focused on operationally defined drawing characteristics in adolescents. These two approaches may provide complementary information about the relationship between TDT features and depression. The aim of this study was to explore the relationship between drawing features in the TDT and depressive disorders in adolescents, with the goal of providing a simple and objective auxiliary screening tool for clinical practice. Studies have shown that individuals with major depressive disorder (MDD) tend to draw trees with significantly smaller crown area, crown height, crown width, trunk width, and overall size compared with healthy controls. Researchers have suggested that the tree crown primarily reflects conscious emotional states, and reduced tree size is consistent with depressive symptoms such as helplessness, anhedonia, and pessimism.^15^ Moreover, certain quantitative indicators of drawing have been found to correlate with the severity of depressive symptoms.^17^, ^18^

In clinical assessment, the TDT is frequently used to evaluate levels of personality functioning.^19^ For example, individuals with neurotic disorders tend to draw a “closed crown,” indicating an effort to establish an integrated self‐representation and clear psychological boundaries.^20^ This pattern contrasts with that observed in patients with thyroid disorders, who more frequently produce “open crowns,” reflecting blurred psychological boundaries.^21^ The TDT has also been shown to be useful in distinguishing between different types of mental disorders, for example, schizophrenia.^22^ Trees drawn by individuals with schizophrenia are typically smaller and often characterized by an “open‐ended trunk,” which has been interpreted as reflecting confusion between self and non‐self or impaired ego functioning.^23^ In contrast, individuals with cognitive impairments (e.g., Alzheimer's disease and mild cognitive impairment) tend to draw even smaller trees with a larger trunk‐to‐crown ratio, accompanied by larger not‐painting periods and less contrast, reflecting progressive impairments in constructive praxis and visuospatial functioning.^24^ Stanzani Maserati et al. further noted that the trunks drawn by individuals with cognitive impairment are typically closed at the top, distinguishing them from the open‐ended trunks commonly observed in schizophrenia.^19^

Research on chronic schizophrenia has found that TDT features (e.g., small size, narrow trunk, open base) show limited ability to distinguish between patients with good versus poor treatment response. These findings suggest that the TDT may reflect relatively stable underlying trait markers rather than state markers that fluctuate rapidly with symptom changes.^23^ Recent empirical studies have also proposed TDT‐based “depression assessment scales,” which have demonstrated high diagnostic efficiency in adolescent populations.^11^ However, research on the relationship between the TDT features and depressive disorder remains limited in China, particularly with respect to large‐sample quantitative analyses focusing on adolescents. Accordingly, the present study aims to investigate the relationship between TDT characteristics and adolescent depressive disorder. By doing so, this study seeks to enrich the methodological framework for depressive disorders screening and to provide a simple, low‐cost, and minimally language‐dependent auxiliary tool for clinical practice, thereby facilitating the early identification and intervention of depressive disorder in adolescents.

### 4.1 Main findings and psychological implications

The results confirmed that adolescents with depressive disorders differed significantly from healthy controls in 10 out of 12 drawing features of the TDT. The depressive group more frequently exhibited features associated with psychological distress, including small trees, shaky lines, trees positioned at the bottom of the page, and scars or blemishes on the trunk.

#### 4.1.1 Tree size and position

In the depressive group, 77.08% of participants drew small trees, compared to only 9.38% in the control group. This aligns with previous studies showing that patients with MDD tend to draw trees smaller than healthy controls.^15^ Small tree size is thought to correspond with feelings of helplessness, lack of interest, and pessimism in depressive symptoms. Additionally, 52.08% of the depressive group placed the tree at the bottom of the page, which in projective tests is often associated with low self‐esteem or a lack of security.

#### 4.1.2 Shading and canopy characteristics

Shading or darkened areas (64.58% in the depressive group) and disordered lines within the tree canopy (79.17% in the depressive group) occurred much more frequently in the depressive group than in controls. In projective theory, shading or dark areas often suggest underlying anxiety, depression, or conflict.^12^ The canopy is considered to reflect the participant's conscious emotional state.^15^ Conversely, the proportion of trees with fruit was significantly lower in the depressive group than in controls (8.33% vs. 28.13%), which may reflect a lack of vitality, sense of harvest, or achievement in real life.^25^

#### 4.1.3 Independent predictors

Binary logistic regression identified three independent predictors significantly associated with adolescent depressive disorders: disordered lines within the tree canopy, detailed tree depiction, and shading or darkened areas. Among these features, disordered lines within the tree canopy showed the largest effect size (OR = 7.828, *p* = 0.003), suggesting a particularly strong association with depressive disorders in the present sample. This factor had the largest effect size and may reflect internal emotional confusion and distress, correlating with clinical symptoms such as intense emotionality, low mental energy, and hesitation.^26^ Such disorganized or agitated drawing patterns suggest that individuals may be experiencing considerable psychological stress or conflict. Adolescent depressive disorders often arise from the interplay of genetic, biological, and environmental factors, and some negative experiences may not be accurately expressed through language or scales, whereas drawing provides a more direct projective medium. This feature showed the strongest predictive effect for depression, indicating its importance as a screening indicator.

Detailed tree depiction was also independently associated with adolescent depressive disorders (OR = 7.094, *p* = 0.007). Previous research has suggested that excessive attention to detail may relate to anxiety, obsessive tendencies, or heightened focus on internal conflict.^25^ It symbolizes the concentration of psychological energy in the subconscious self, emphasizing internal experiences over external reality. This aligns with ruminative thinking observed in depressive patients, who often overthink negative experiences, leading to persistent sadness and distress. This feature demonstrated strong predictive value in adolescents with depression.

Shading or darkened areas constituted the third independent predictor of adolescent depressive disorders (OR = 4.155, *p* = 0.045). This further supports its role as a projection of depressive tendencies. Previous studies have shown that individuals experiencing anxiety or depression often add shading to the trunk or branches,^12^ reflecting negative emotions such as pessimism. This confirms that this feature can reflect an individual's emotional state.

These findings are consistent with earlier studies linking quantitative TDT measures, such as size, with depression severity.^14^

The study also examined “trees with sharp, knife‐like branches” and “trees with fruit.” Prior research suggested that knife‐like branches may project restlessness or conflict,^23^ indicating aggression or irritability, while fruit is typically interpreted as a symbol of hope or goals and corresponds to an individual's connection with their environment and personal growth and aspirations.^27^ However, these two features did not reach statistical significance in this study. Possible explanations include a small sample size, reducing statistical power, and some participants not clearly distinguishing between “fruit trees” and “trees,” leading to underrepresentation of fruit. Nevertheless, the psychological relevance of these features warrants further investigation.

### 4.2 Potential supplementary value of the TDT

Depressive disorder is one of the most common psychiatric conditions in adolescents, with a globally increasing prevalence. However, up to 80% of adolescents with depression do not receive appropriate care or follow‐up assessment. Universal screening is a feasible approach to bridge this clinical care gap and improve diagnostic rates.

Based on the three independent TDT predictors, the logistic model was constructed and demonstrated good predictive performance, with an overall accuracy of 83.8% and an AUC of 0.900. This indicates that the TDT may provide valuable auxiliary support in screening and diagnosing adolescent depressive disorders.^15^

Although self‐report scales (e.g., SDS) are convenient, their accuracy can be affected by subjective responses and social desirability, potentially reducing sensitivity. As a low‐cost, language‐independent projective drawing test, the TDT can more directly reflect underlying psychological states. The TDT bypasses language barriers and captures deep emotional and cognitive structures through unconscious drawing behavior (e.g., disordered canopy, shading, or small trees), allowing clinicians to more objectively identify and assess hidden depressive traits. Therefore, in primary care or school settings, the TDT, combined with our logistic model, can serve as a supplement to self‐report scales or a preliminary screening tool, helping to identify high‐risk individuals early and promoting timely interventions.

Despite the small sample size, the quantitative analysis provides an empirical basis for conducting TDT research in Chinese adolescents. Drawing features may represent “trait markers,” reflecting stable underlying psychological states.

## 5 LIMITATIONS

This study has several limitations. The relatively small sample and single‐area recruitment may limit the generalizability of the findings. In addition, the two groups completed the TDT under different testing settings and administration formats, which may have influenced drawing behavior. The binary coding of drawing characteristics also reduced continuous information, such as variations in tree size and shaded area. Finally, the predictive model was developed and evaluated in the same cross‐sectional sample without independent or prospective validation. Therefore, the findings should be considered exploratory and require confirmation in larger, multicenter, and prospectively validated samples.

## 6 CONCLUSION

This study indicates that adolescents with depressive disorders exhibit specific drawing features in the TDT, among which “trees with shading or darkened areas,” “trees depicted in a detailed manner,” and “disordered lines within the tree canopy” demonstrate strong predictive power. The model based on these features shows good discriminatory ability, suggesting that the TDT reveals underlying psychological conflicts and emotional states. However, the TDT should not be regarded as an independent screening or diagnostic tool. Future studies should employ larger, multicenter, longitudinal designs to further validate its clinical utility and integrate intelligent, objective assessment methods, promoting standardized application of the TDT in mental health screening and intervention.

## AUTHOR CONTRIBUTIONS

Yibin Lu and Xutong Chen contributed to the conceptualization, methodology, and investigation. Qiqige contributed to formal analysis and writing of the original draft. Rong Yi contributed to the original drafting and revision. Jike Bai contributed to conceptualization, supervision, and project guidance. All authors reviewed and approved the final manuscript.

## CONFLICT OF INTEREST STATEMENT

The authors declare no conflicts of interest.

## ETHICS STATEMENT

This study was approved by the Ethics Committee of Dongguan Seventh People's Hospital (Approval No. 2022‐003). This study was conducted in accordance with the local legislation and institutional requirements. Written informed consent was obtained from all participants and their legal guardians prior to participation.

## Data Availability

All original data are available from the corresponding author upon reasonable request.
